# Discharge against Medical Advice (DAMA) from an Emergency Department of a Tertiary Care Hospital in Saudi Arabia

**DOI:** 10.1155/2019/4579380

**Published:** 2019-11-28

**Authors:** Ashraf El-Metwally, Nesreen Suliman Alwallan, Ali Amin Alnajjar, Nida Zahid, Khalid Alahmary, Paivi Toivola

**Affiliations:** ^1^College of Public Health and Health Informatics, King Saud Bin Abdulaziz University for Health Sciences, Riyadh, Saudi Arabia; ^2^King Abdullah Bin Abdulaziz University Hospital, Princess Nourah University, Riyadh, Saudi Arabia; ^3^Aga Khan University, Karachi, Pakistan; ^4^Department of Health Systems Management, College of Public Health and Health Informatics, King Saud Bin Abdulaziz University for Health Sciences, Riyadh, Saudi Arabia; ^5^King Abdullah Specialist Children's Hospital, King Abdulaziz Medical City, Riyadh, Saudi Arabia

## Abstract

**Background:**

The discharge against medical advice (DAMA) in the Emergency Department (ED) is an issue of great concern because it may result in adverse consequences at a later stage. The reported worldwide prevalence of DAMA ranges from 0.07 to 20% for emergency admissions. The outcomes of DAMA can have significantly damaging effects, causing possible relapses of disease, readmission, and increases in medical costs for the patient. Therefore, it is imperative to identify the predictors of DAMA in ED.

**Methods:**

It was a cross-sectional study. The medical records used were those of all the patients (*n* = 11513) admitted to the Emergency Department (ED) of King Abdullah Bin Abdulaziz University Hospital (KAAUH) in Riyadh, Saudi Arabia, between 2017 and 2018. A thorough analysis was performed using IBM SPSS Statistics version 22. Descriptive statistics were reported for quantitative and categorical variables and assessed by independent *t*-test/chi-square/ANOVA (analysis of variance), where appropriate. Unadjusted and adjusted odds ratios with their 95% CI (confidence interval) were reported by performing logistic regression. A *p* value of ≤0.05 was considered statistically significant throughout the study.

**Results:**

The prevalence of DAMA in our study was 1%. In a multivariable analysis, after adjusting for the other covariates, we observed a significant interaction between age and gender. It was observed that the odds of DAMA for ≤40-year-old males were 3.12 times higher than those of a ≤40-year-old female (*p* value < 0.1). To further investigate this interaction, men and women were modeled separately in multivariable models using the same covariates. We found that, for men, the effect of age (≤40 years) was significant (OR = 3.94, 95% CI 1.31–11.80, *p*=0.014), while, for women, the effect of age (≤40 years) was not as pronounced (OR = 1.27, 95% CI = 0.66–2.42, *p*=0.27).

**Conclusions:**

Our study concluded that DAMA was more likely among younger male patients (≤40 years of age). Most of the patients with DAMA were presented to the urgent care of the Emergency Department. We recommend that patients be given some financial support to bear the expenses of the hospital stay from the healthcare facility or from the state. Future studies should assess the socioeconomic status of the patients and estimate the cost that is incurred by the patients.

## 1. Background

The Emergency Departments (ED) of hospitals play a crucial role in preserving people's lives [1]. DAMA from Emergency Departments is a quite concerning issue because it is assumed that these patients are leaving too soon and adverse consequences may very likely follow their discharge [2]. DAMA has a clearly negative impact on treatment outcomes and healthcare resource utilization in addition to exposing healthcare providers to the hazards of proceedings. Moreover, DAMA is associated with higher rates of readmission for the same diseases for which patients were initially admitted or for related morbidity of the diseases, which could lead to a high long-term financial cost of medical care [3].

The reported worldwide prevalence of DAMA for general medical admissions ranges from 0.7% to 2.2% and from 6% to 54% for psychiatric admissions and is 0.9% for emergency admissions. Various studies suggest a positive correlation between DAMA and male gender, and DAMA and increasing age [2, 4, 5]. Predictors of DAMA such as younger age, male sex, substance abuse disorders, lack of a personal physician, and lack of health insurance have also been reported in the literature [2]. A study from Nigeria reported that the decision of DAMA was made mainly by patients' relatives (58.4%) followed by patients themselves (40.7%). This shows that the opinion of family members plays an important role in an individual's healthcare [3]. DAMA exposes the patient to the risk of an inefficiently treated medical problem and could very easily lead to readmission and extended morbidity. A study [6] found that from the patients who were DAMA, 32% were readmitted within 30 days, while only 12% of patients who underwent regular discharges had readmission within 30 days. Moreover, patients who were DAMA were more likely to have readmission for the same (or related) diseases in the following month (28% vs. 8%) and had longer length of hospital stays for any readmission (median 5 vs. 0 days) [7]. In addition, the relationship between healthcare providers and the patients may be strained due to readmission caused by DAMA. It is essential to understand the factors that cause DAMA and it is of immense importance to identify those at higher risk of DAMA to avoid excess morbidity, mortality, and healthcare costs [6].

A study conducted in Saudi Arabia reported the prevalence of DAMA and its associated factors among patients with acute myocardial infarction [8]. However, to the best of our knowledge, no study has been reported from this region reporting the prevalence of DAMA and its predictors among patients presenting to the Emergency Department of a tertiary care hospital. The outcomes of DAMA can have extremely detrimental effects on a patient's situation, even up to the point of death, as well as associated side effects, which cannot be cured, causing relapses, readmissions, and increases in medical costs for the patient. Therefore, it is imperative to identify the predictors of DAMA in ED. In the light of literature, the objective of the study was to determine the prevalence of DAMA among adult populations presenting to a tertiary care hospital in Saudi Arabia. The study aimed to further assess the predictors of DAMA among that same population.

## 2. Materials and Methods

It was a cross-sectional study design. All patients were admitted to the Emergency Department (ED) of KAAUH in Riyadh, Saudi Arabia, in the period between January 2017 and March 2018. The ED offers medical care to all students enrolled at Princess Nourah University (PNU) and employees of the university and the hospital as well as their families (approximately 70,000). The medical records used were composed of 11513 patients admitted to the adult ED.

KAAUH is a major academic governmental hospital funded by the Ministry of Education. It is composed of 300 beds and has seven departments that offer healthcare to patients with all types of diseases. It has 900 employees, 113 consultants, 320 residents, and 320 nurses. The ED of the hospital was opened in March 2017 and is composed of the following sections: Acute Care, Triage Unit, and Resuscitation Unit. The ED manages approximately 47 admitted cases per day.

As per regulation and policies related to the hospital ED, the information related to all patients is entered on the Health Information Management System-Track Care and the information entered for each admitted patient includes the following: gender, age at admission, triage category, discharge date and time, bed, and discharge classification. Data from the system was extracted and entered into an Excel sheet for data cleaning, and basic exploratory analysis was performed prior to SPSS data entry.

Ethical approval was taken from an ethical review committee. Approval was also taken from KAAUH to retrieve information on patients from their medical records. An analysis was performed using IBM SPSS version 22. Descriptive statistics were computed for categorical variables, such as age, gender, and triage, by computing their frequencies and percentages. Its relationship with discharge status was then assessed by chi-square test. The quantitative variable, such as age, was computed by their mean ± S.D and its relationship with discharge status was assessed by independent *t*-test/ANOVA, where appropriate. Unadjusted and adjusted odds ratio with 95% CI were reported by performing logistic regression modeling. A *p* value of ≤0.05 was considered statistically significant throughout the study.

## 3. Results

### 3.1. Description of the Population

Table 1 shows the description of the study participants presenting to the ED of a tertiary care hospital in Saudi Arabia. The mean age of the study's participants was 34.79 ± 12.37. A higher proportion of participants were less than 40 years of age (73.9%) while 26.1% were greater than 40 years of age. A higher proportion of females than males presented to the ED, i.e., 7821 (70.4%) versus 3293 (29.6%). The prevalence of DAMA was 1%. About 36.2% were sent home, followed by 8.3% admitted to the hospital, 0.9% were discharged with outpatient appointments, and 0.5% were transferred to another healthcare facility. We observed that a higher proportion (60.9%) of patients presented to the less urgent care of ED followed by urgent triage (24.5%), nonurgent triage (13.9%), and emergent triage (0.5%), while non-ED patients were 0.2%.

### 3.2. Relationship of Demographic Factors and Triage with Discharge Status

Table 2 presents the relationship of discharge status and demographic factors among the adult population presenting to the ED of a tertiary care hospital in Saudi Arabia. There was a significant relationship between age and discharge status (*p* value < 0.001). We observed that a higher proportion of patients (62.7%) ≤ 40 years of age were DAMA as compared to those who were more than 40 years of age (37.3%). The mean age was higher among those who got DAMA (37.51 ± 14.17) as compared to those who were admitted to the hospital, discharged with outpatient appointment, or sent home. However, there was no significant difference observed between the mean age of DAMA patients and patients with other discharge statuses on post hoc analysis. We found a significant relationship between gender and discharge status of the patients (*p* value < 0.001). We also found a significant relationship between triage and the discharge status of the patients (*p* value < 0.001). We noted that the majority of the DAMA patients were of urgent triage (49.1%) followed by those of less urgent (45.6%) triage, nonurgent (3.5%) triage, and finally emergent (1.8%) triage. Similarly, a higher proportion of patients who were admitted to the hospital and were transferred to another healthcare facility presented to urgent care (76.1% and 48.3%, respectively), while those who were sent home and were discharged to outpatient facilities presented to less urgent facilities (64.5% and 54%, respectively).

### 3.3. Relationship of Demographics and Triage with DAMA

Table 3 presents the relationship of DAMA with the demographics and triage. We observed that a high proportion of patients with DAMA (62.7%) were less than or equal to 40 years of age as compared to those who were >40 years (37.3%) (*p* value = 0.05). There was a significant relationship between triage and DAMA; we observed that a higher proportion of patients who requested DAMA presented to urgent care (49.1%) as compared to those who did not request DAMA (24.6%). However, there was no significant relationship of gender and DAMA (*p* value > 0.05).

### 3.4. Univariate and Multivariable Analysis

Table 4 shows the univariate and multivariable analyses for assessing the relationship of DAMA with demographics and triage. In a univariate analysis, age and triage were significant at *p* value < 0.2. We observed that the odds of DAMA among patients ≤40 years were 1.67 times greater when compared to those of patients >40 years of age (95% CI = 0.99–2.85). In the multivariable analysis, after adjusting for the other covariates, we found a significant relationship between age and gender. It was observed that the odds of DAMA for ≤40-year-old male patients were 3.12 times higher than those of a ≤40 years old female patient (*p* value < 0.1). To further investigate this interaction, men and women were modeled separately in multivariable models using the same covariates. We observed that for men the effect of age (≤40 years) was significant (OR = 3.94, 95% CI 1.31–11.80, *p*=0.014), while for women the effect of age (≤40 years) was not significant (OR = 1.27, 95% CI = 0.66–2.42, *p*=0.27). This confirms the multivariable analysis finding that the odds for DAMA increased significantly in men with age (≤40 years), but not in women.

## 4. Discussion

In our study, the prevalence of DAMA among patients presenting to ED was 1% over a period of 1 year. A study from Spain reported a prevalence of DAMA from ED ranging from 0.07% to 0.7% [9]. Moreover, another study from a tertiary care hospital in Iran revealed a DAMA rate in ED of about 20.0%. [2]. A study from USA reported a prevalence of DAMA from ER of 0.1 to 2.7% [10]. Therefore, our results suggest that the prevalence of DAMA in our region might be comparable with that in other regions of the world.

In this study, we discovered a significant association of age with DAMA. DAMA was more likely among the younger patients, which is consistent with the findings from other studies [2, 4, 5]. A study from Pakistan reported that the highest rate of DAMA was found among a younger age group [11]. Moreover, a systematic review, which was carried out on 61 studies, showed that young age is indeed a predictor of DAMA [12]. A study conducted at the Mayo Clinic also revealed that the trend for DAMA was higher among younger individuals [6]. In addition, another study from Iran reported that gender had a significant relationship with discharge status and the DAMA rate among men was 47% greater than that of women [13]. In our study, we also noticed a significant correlation between age and gender. It was observed that DAMA was more likely among younger males (≤40 years) compared to females of the same age group. As reported in the literature, financial problems and fears about handling life can be factors affecting DAMA. In this regard, men of a younger age group, who are traditionally the main breadwinners and are more likely to have a greater financial burden, prefer to take DAMA more than some women. The results of the studies conducted in America and Iran also confirm the role of gender in predicting DAMA [12, 14].

Moreover, a higher proportion of patients with DAMA presented to urgent care. Usually patients presenting to less urgent care at ED are those who are nonemergency cases and thus have shorter stays at the hospital. On the other hand, emergency cases are usually admitted at the urgent care and are advised to stay longer at the hospital due to their critical condition. Long stays at the urgent care would increase the total expenditures of the patients, which might be difficult for the patients to bear. Therefore, in such situations, the patients would prefer DAMA and we anticipate that this might be one of the main reasons for DAMA. There were several strengths to our study, the most important of which being that, to the best of our knowledge, this was the first study conducted at Saudi Arabia assessing demographic factors of DAMA at the ED. Since this tertiary care hospital caters to people with different demographic, our study results can be generalized to the Emergency Department of all the tertiary care hospitals in Saudi Arabia.

However, there were certain limitations to our study. Since the data had been collected from the medical records at the hospital, there was some missing data. It is crucial for future research to investigate what were the risk factors for DAMA. We have only been able to investigate risk factors in terms of age and gender. Several other risk factors may be important, for example, course of DAMA, disease categories, and types of admission. Moreover, the information on the socioeconomic factors (such as educational status, occupation factors, and monthly income) and reasons for DAMA were not available on the system, which might be an important predictor of DAMA at the Emergency Department. This might be an important research question for future studies in Saudi Arabia.

## 5. Conclusions and Recommendations

The conclusion of our study was that gender and age interact in the prediction of DAMA. While larger, prospective studies are needed to validate these findings, this study represents a first step toward mechanistic investigations to lay the foundation for the development of sex-tailored therapies. However, we found that among males >40 years of age DAMA was significantly higher. Majority of the patients with DAMA presented to urgent care of ED. We recommend that patients should be given some financial support to bear the expenses of the hospital stay from the healthcare facility or from the state. Moreover, future studies should assess the socioeconomic status of the patients and a cost analysis should be performed to understand how much cost is incurred by patients presenting to the ED department.

## Figures and Tables

**Table 1:** Description of the study participants.

Variables	Frequency	Percent (%)
Age (mean ± SD) (in years)	34.79 ± 12.37	

*Age (in years)*		
≤40	8497	73.9
>40	3008	26.1
Total	11505	

*Gender*		
Male	3293.00	29.6
Female	7821.00	70.4
Total	11113	

*Discharge classification*		
Discharge against medical advice	59.00	1
Admission to hospital	497.00	8.3
Home	5357.00	36.2
Discharged with outpatient appointment	55.00	0.9
Transfer to another healthcare facility	30.00	0.5
Total	5998	

*Triage category*		
Emergent	56.00	0.5
Urgent	2682.00	24.5
Less urgent	6676.00	60.9
Nonurgent	1521.00	13.9
Non-ER patient	22.00	0.20
Total	10955	

**Table 2:** Relationship of discharge status and demographic factors and triage among adult population.

Variables	Discharge against medical advice	Admission to hospital	Home	Discharged with outpatient appointment	Transfer to another healthcare facility	*p* value
Age in years (mean ± SD)	37.53 ± 13.17	34.53 ± 12.51	34.64 ± 12.03	41.05 ± 13.26	44.81 ± 13.11	<0.001^*∗*^

*Age in years*						<0.001^*∗*^
≤40 years	37 (62.7%)	392 (78.9%)	3955 (73.8%)	25 (45.5%)	12 (40%)	
>40 years	22 (37.3%)	105 (21.1%)	1402 (26.2%)	30 (54.5%)	18 (60%)	
Total	59	497	5357	55	30	

*Gender*						<0.001^*∗*^
Female	45 (76.3%)	443 (89.1%)	3851 (71.9%)	35 (63.6%)	20 (67.7%)	
Male	14 (23.7%)	54 (10.9%)	1506 (28.1%)	20 (36.4%)	10 (33.3%)	
Total	59	497	5357	55	30	

*Triage*						<0.001^*∗*^
Emergent	1 (1.8%)	16 (3.5%)	8 (0.2%)	0	7 (24.1%)	
Urgent	28 (49.1%)	343 (76.1)	1024 (19.9%)	17 (34%)	14 (48.3%)	
Less urgent	26 (45.6%)	91 (20.2%)	3321 (64.5%)	27 (54%)	6 (20.7%)	
Nonurgent	2 (3.5%)	1 (0.2%)	781 (15.2%)	6 (12%)	2 (6.9%)	
Non-ER patient	0	0	12 (0.2%)	0	0	
Total	57	451	5146	50	29	

^*∗*^Significant at *p* value ≤ 0.05 by using ANOVA test/chi-square test.

**Table 3:** Relationship of discharge against medical advice (DAMA) with demographic factors and triage.

Variables	Discharge against medical advice (DAMA)		*p* value
	Yes (*n* = 59)	No (*n* = 5939)	
Age in years (mean ± SD)	37.53 (13.178)	34.74 (12.13)	0.08

*Age*			0.05^*∗*^
≤40 years	37 (62.7%)	4384 (73.8%)	
>40 years	22 (37.3%)	1555 (26.2%)	

*Gender*			0.60
Female	45.0 (76.3%)	4349 (73.2%)	
Male	14.0 (23.7%)	1590 (26.8%)	

*Triage*			<0.001^*∗*^
Emergent	1 (1.8%)	31.0 (0.5%)	
Urgent	28 (49.1%)	1398 (24.6%)	
Less urgent	26 (45.6%)	3445 (60.7%)	
Nonurgent	2 (3.5%)	790 (13.9%)	
Non-ER patient	0 (0.0%)	12 (0.2%)	

^*∗*^Significant at *p* value ≤ 0.05 by using independent test/chi-square test/Fisher's exact test.

**Table 4:** Univariate and multivariable analyses for assessing relationship of DAMA with demographics and triage.

Variables	Unadjusted OR (95% CI)	Adjusted OR (95% CI)
Age in years	0.98 (0.96–1.002)^*∗*^	—

*Age*		
≤40 years	1.67 (0.99–2.85)^*∗*^	0.68 (0.29–1.61)
>40 years (ref)	1	1

*Gender*		
Male	1.18 (0.64–2.15)	1.27 (0.66–2.42)
Female (ref)	1	1

*Triage*		
Urgent	1.61 (0.212–12.2)^*∗*^	—
Less urgent	4.27 (0.56–32.49)	
Nonurgent	12.74 (1.13–144.3)	
Non-ER patient emergent (ref)	—	
	1	

*Age, gender*		3.12 (0.86–11.12)^*∗*^
≤40 years, male	—	3.94 (1.31–11.80)^*∗*^
≤40 years, female		1.27 (0.66–2.42)

^*∗*^Significant at *p* value < 0.01.

## Data Availability

The datasets analyzed/generated during the current study are not publicly available due to patient confidentiality.

## Abbreviations

DAMA: Discharge against medical advice
ED: Emergency Department
KAAUH: King Abdullah Bin Abdulaziz University Hospital
ANOVA: Analysis of variance
CI: Confidence interval
PNU: Princess Nourah University.
